# Acoustic levitation with optimized reflective metamaterials

**DOI:** 10.1038/s41598-020-60978-4

**Published:** 2020-03-06

**Authors:** Spyros Polychronopoulos, Gianluca Memoli

**Affiliations:** 1 University of Sussex, School of Engineering and Informatics, Brighton, BN1 5EL United Kingdom; 2 Present Address: National and Kapodistrian University of Athens, Department of Informatics and Telecommunications, Athens, Greece

**Keywords:** Computational methods, Acoustics

## Abstract

The simplest and most commonly used acoustic levitator is comprised of a transmitter and an opposing reflecting surface. This type of device, however, is only able to levitate objects along one direction, at distances multiple of half of a wavelength. In this work, we show how a customised reflective acoustic metamaterial enables the levitation of multiple particles, not necessarily on a line and with arbitrary mutual distances, starting with a generic input wave. We establish a heuristic optimisation technique for the design of the metamaterial, where the local height of the surface is used to introduce delay patterns to the reflected signals. Our method stands for any type and number of sources, spatial resolution of the metamaterial and system’s variables (i.e. source position, phase and amplitude, metamaterial’s geometry, relative position of the levitation points, etc.). Finally, we explore how the strength of multiple levitation points changes with their relative distance, demonstrating sub-wavelength field control over levitating polystyrene beads into various configurations.

## Introduction

Since the first levitation of an object with sound, almost a century ago, the physics behind levitation and manipulation of objects with acoustic waves has been thoroughly studied^1–3^. With the exception of a limited number of studies involving chemical analysis^4,5^, acoustic levitation has been mainly used to observe the dynamics of levitated objects^6–8^, including small animals^9^. More recently, acoustic levitation in air has been used to display technical information^10,11^, to convey graphical messages^11,12^ or to elicit novel interactions^13,14^ and multi-sensory experiences^15^.

The standard acoustic levitator involves the creation of a standing wave^16–19^, either by two opposing ultrasonic transducers or a source and a reflector. In air, particles in a levitator would aggregate at the nodes of the field (i.e. the low-pressure points), which are typically arranged in a line between the elements, at distances of half of the wavelength of the emitted signal. The ability to freely position objects in mid-air seems therefore limited to multiples of half of a wavelength ($\lambda /2$) in the vertical direction^19–21^. Conversely, acoustic self-aggregation of particles into structures with mutual distances much smaller than $\lambda /2$ has been observed experimentally in horizontal standing waves, both in air^22–24^ and in water^25,26^. This phenomenon, due to particle-particle interactions^26,27^, requires however particles much smaller than the wavelength ($\sim \lambda /100$): overcoming this limit in acoustic levitators – which tend to use larger particles – requires a finer control on the acoustic field.

More control on the field (e.g. focusing the acoustic energy in specific locations) can be achieved using Phased Arrays of ultrasonic Transducers (PATs)^10,12,28–31^. In these setups, the position of the levitated object can be modified by adjusting either the transducers’ phases^21,31^ or amplitudes^32–34^. Multi-object levitation in predefined positions can thus be achieved, either by static^15,35–37^ or multiplexing^38^ PATs. Using PATs adds lateral control, as shown by Ayumu Watanabe *et al*.^21^, who used a PAT and a flat reflecting surface to create two non-synchronous levitation points at a horizontal distance of $\lambda$, but the limitation of $\lambda /2$ in the vertical direction still stands. Furthermore, PATs require elaborate and expensive electronics and, since often the commercial ultrasonic transducers’ diameter is much greater than half of a wavelength, they result in a poor spatial resolution of the created field.

With the advent of acoustic metamaterials^39^, which allow phase engineering on impinging sound waves using unit elements with a surface area smaller than the one of the corresponding transducers, the sound-field can be controlled with greater spatial resolution than when traditional sources (e.g. transducers) are used alone^40,41^. Using this method, higher control on the field can therefore be achieved between the source and a reflecting metamaterial, resulting in effects like self-bending beams^42^, broadband extraordinary reflection^43^ and even unidirectional transmission^44,45^. Zhu *et al*.^46^ pioneered an even greater control on the field by tailoring the local loss in the reflecting metamaterial, thus modifying both phase an amplitude of an input wave at normal incidence. Recent studies propose ways of controlling the scattering behaviour of reflective metamaterials with very simple geometries^47^ to achieve control even at oblique incidence, including using only two types of unit cells^48^ and Archimedean spiral structures^49^. To the author’s best knowledge, however, Melde *et al*.^20^ were the only ones to use a reflecting metamaterial for acoustic levitation, managing to suspend two lines of water droplets, with vertical distances of $\lambda /2$ between the droplets in each line and a horizontal distance of $2\lambda$ between the lines.

It is worth noting that all the aforementioned studies with reflective metamaterials assume a plane wave input, which was achieved using a single source^20,46^ in the far field. In particular, in Melde *et al*.^20^ the transducer emits a sine wave of 100 kHz ($\lambda \sim 3.4\,{\rm{mm}}$) with a control space between the sound source and the metamaterial of 25.7 mm and in Zhu *et al*.^46^ the source emits a sine wave of 17 kHz ($\lambda \sim 20\,{\rm{mm}}$) and is located 3 m away from the metamaterial’s surface. These solutions limit the strength of the field and the scalability of the control volume.

Here we propose a computational method to shape the acoustic field and create multiple traps in predefined positions, based on a generic source and a reflective acoustic metamaterial. Here, the metamaterial is comprised of a grid of surfaces at different heights, each one of them introducing a unique delay to the reflected wave and shaping the sound-field – between the source and the reflector – as required. Our method holds for a system with multiple degrees of freedom (i.e. phase, amplitude and relative position of the transducers, geometry of the metamaterials or the relative position of a mesh of objects to be levitated). We apply our method to demonstrate a new type of acoustic levitator, based on a simple single-phased array of transducers and a reflective acoustic metamaterial, capable of levitating objects ($\sim \lambda /4\,$in size) even at distances non-multiples of $\lambda /2\,$in the vertical direction.

## Results

### Computational operation

**Figure 1:**
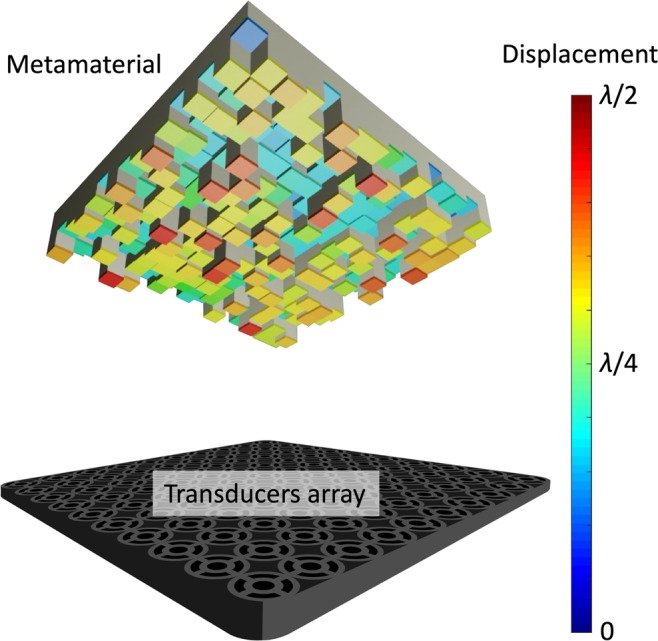
A schematic representation of the system described in this work, highlighting the transducers’ array (at the bottom) and the reflective metamaterial (on top) with unit cells of variable heights (displaced from 0 to $\lambda /2$), as in the colour bar. Image created using Autodesk 3ds Max 2018 and Adobe Illustrator CC 2017.0.2.

To maintain the most generic case, we will consider a generic levitator to be comprised of one or more ultrasonic sources, distributed in a surface, and of an opposing acoustic metamaterial, which acts as a reflector. The transducers can be either single-phased or, for a system with more degrees of freedom, arranged in a PAT. The metamaterial’s elements are displaced at various heights from the metamaterial’s datum (see Fig. 1), creating a distribution of delay paths of reflecting waves. To calculate the acoustic pressure *p* in a generic point ($x,y,z$) between the emitters and the metamaterial, we consider the direct and the reflected signal, as in Eq. (1):

1 $${p}_{(x,y,z)}=\mathop{\underbrace{{p}_{d(x,y,z)}}}\limits_{direct\,signal}+\mathop{\underbrace{{p}_{r(x,y,z)}}}\limits_{reflected\,signal},$$

where the pressures include the contributions of all the transducers and the elements of the metamaterial. Further details on the calculation of the pressure field are described in the supplementary information (S1), but it should be noted that, in our simulations, terms higher than first order reflections could easily be neglected (i.e. second order reflections are approximately three orders of magnitude smaller than the first one, as shown in the supplementary information, S2). Equally, we neglect the coupling (“cross-talking”) between the different parts of the metasurface. In doing so, we neglect the transmission of energy from air to the metamaterial structure: an assumption justified by the ultrasonic frequencies and the difference in impedance between air and the metasurface material.

For our simulations, we consider a source comprised of multiple transducers. In the most common scenario, considering the dimensions of the current transducers available in the market, the diameter of the ultrasonic emitters is larger than the wavelength of the transmitted signal and the transducers cannot be placed in a tight mesh. In this case, the output sound profile of an array of ultrasonic transducers is not comparable to a plane wave (see supplementary information, S7. Fig. S6a) and therefore, the direct signal ${p}_{d}$ Eq. (1) is calculated by the summation of all the transducer signals^50^, each one of them approximated as circular piston source^51^. We assume the source to be placed in front of a rectangular reflective metamaterial (see Fig. 1), comprised of square unit cells of variable height, each with the same lateral dimensions. Similarly, the reflected signal is calculated by the summation of the reflected signals ${p}_{r}$ Eq. (1) on the metamaterial’s grid of reflectors simulated as square piston sources^51^.

**Figure 2:**
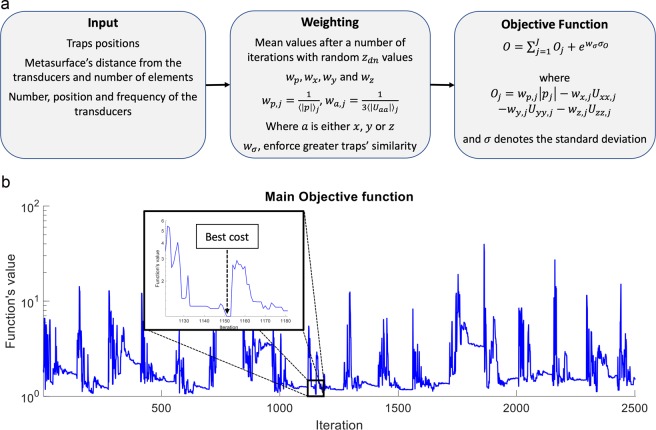
(**a**) Block diagram of the calculation of the objective function. (**b**) SA performance example for 2500 iterations minimizing the objective function where the best cost is at iteration 1480. The example shown here is related to the creation of two traps with a horizontal distance in between them of $2\lambda$ at a vertical position in the middle of the distance between an 8 × 8 array of transducers (10 mm in diameter) operating at 40 kHz and a metasurface comprised of 16 × 16 unit cells, each $\lambda /2\,\times \lambda /2\,$in lateral size (approximately 4.3 mm by 4.3 mm). Here, ${z}_{dn}$ are the heights of the single unit cells over the metasurface’s datum.

In order to create levitation conditions (i.e., “traps”) at predefined points in the space between the transducer and the metamaterial (i.e. the “cavity”), the pressure at these locations needs to be minimized, while simultaneously finding a stable value of the acoustic force^28^ (i.e., a zero for its gradient, related to the Laplacian of the Gor’kov’s potential $U$). The minimization of the objective function (Eq. (2)) is fulfilling the above requirements for multiple points ($j=1,2,\ldots ,J$). For nonlinear problems where finding an approximation of the global optimum is more important than finding a more precise local optimum, a simulated annealing (SA)^52^ stochastic optimizer is preferable to alternatives, such as gradient descent^52^. Therefore, in our method we incorporated SA to minimize the objective function

2 $$O=\mathop{\sum }\limits_{j=1}^{J}\,[{w}_{p,j}|{p}_{j}|-\,{w}_{x,j}{U}_{xx,j}-{w}_{y,j}{U}_{yy,j}-{w}_{z,j}{U}_{zz,j}]+{e}^{{w}_{\sigma }{\sigma }_{O}},$$

where $J$ is the number of the traps, ${p}_{j}$ is the pressure at the traps’ position, $U$ is the Gor’kov’s potential energy^1^, with ${U}_{xx}$, $\,{U}_{yy}$ and $\,{U}_{zz}$ being its second order derivatives of the potential over $x\,,y$ and $z$ coordinates. Equation (2) also includes the relative weighting factors for the absolute pressure and the derivatives of the potential energy – respectively ${w}_{p},\,{w}_{x},\,{w}_{y}$ and ${w}_{z}$ – that were used in the optimisation. In order to ensure that the traps are of similar quality the objective function includes the exponential of the standard deviation of all the individual traps’ objective functions (${O}_{j}$, as defined in Fig. 2a) multiplied by a weighting factor (${w}_{\sigma }$) to enforce greater similarity (see supplementary information, Sections S5 and S6).

These numerical assumptions reflect how our levitator works. In a standard levitator, the wave from the single reflecting surface has a singular phase resulting in a standing wave sound-field. In our case, since the metamaterial comprises multiple surfaces at various heights, we can achieve a phase distribution of multiple reflected waves. These add-up to the direct field (coming from the source). Schematically, the number of the created standing waves for such a system, with only 1st order reflections (only on the metamaterial’s surface) considered, are the number of sources multiplied by the number the metamaterial’s elements.

It is worth noting that Eq. (2) depends on how we modelled the interactions between the particle and the field i.e. on the formulation of the potential energy. While we included in the potential both the acoustophoretic (monopole) and the scattering (dipole) term for a sphere, according to classical Gor’kov formulation, we neglected particle-particle interactions (quadrupole). As observed by other authors^26,27^, in the case of a sinusoidal field (like the one in a standard, 1D levitator) particle-particle interactions scale with the ${\xi }^{-4}$ ($\xi$ is the mutual distance between the centre of the particles) and generate forces which are repulsive in the direction of variation of the wave (typically the same as gravity, in a classical 1D levitator) and attractive in the perpendicular plane. According to our evaluations (based on the 1D case and particles with a maximum diameter of $\lambda /2$) adding particle-particle interactions would contribute a maximum of 5% to the force balance in the direction of gravity (see methods). Interactions would therefore not be sufficient to influence the position of the traps but may act as de-stabilising factor while attempting to levitate multiple particles along gravity. Similarly, in the horizontal plane they may force coalescence for two particles levitated sufficiently close.

Equally important is the fact that we used a formulation of the scattered field valid for particles much smaller than the wavelength (i.e. Rayleigh approximation). The next approximation (i.e., Mie scattering^53^) is beyond the scope of this study, but using the results by Silva *et al*.^54^ we estimated that, in the 1D case and for the particles used in this study (i.e. radii below 0.12$\lambda$) the radiation force is reduced by a maximum of 17% (relative to the Rayleigh case). The position of the traps would not change, but the force would scale accordingly (see supplementary information, S3).

The three steps to form the objective function are shown in Fig. 2a. First, the user selects the traps positions where the particles will levitate, the metamaterial’s datum height above the array of transducers, its surface area and number of elements, the number and arrangement of transducers and their emissions frequency are defined. Subsequently, the weighting factors are calculated by running several iterations with a random set of variable values. Here, this step includes as variables the vertical displacements of the metamaterial’s elements ${z}_{dn}$ relatively to the metamaterial’s datum. At the final step, the objective function is formed and then introduced to the optimisation algorithm (SA). The algorithm will try one set of values for the variables at every iteration in order to minimize the objective function and output an optimal set and thus try fulfilling the levitation conditions. It should be noted that the optimizer may fail to come back with positive results. This may happen if the system does not meet the requirements to create levitation points at the defined points (e.g. if the metamaterial’s elements are not sufficient to create the desirable wavefront) or simply because the number of iterations was not sufficient. In order to optimize the random reflectors’ displacement values that SA generates at every iteration and more efficiently track down the optimum set of candidates, we applied a weighting factor to every variable (reflecting surface). The weighting of each element is calculated by considering its significance to create the desirable acoustic field and it is introduced in the optimizer to denote the probability to alter the relative variable in the next iteration (see supplementary information, Section S6).

Figure 2b reports an example of this procedure, relative to the creation of two traps at a mutual horizontal distance of $2\lambda$ and at a vertical position mid-way between an 8 × 8 array of transducers (10 mm in diameter, operating at 40 kHz) and a metamaterial comprised of 16 × 16 unit cells, each $\lambda /2\,\times \lambda /2$ in lateral size (approximately 4.3 mm by 4.3 mm). Figure 2b shows a plot of the first 2500 iterations of the objective function, operating with 256 variable heights (${z}_{dn}$), with the best condition found (best cost) at iteration number 1152. We found that in order to guarantee good results for two levitation points (e.g. in the examples presented in this work), the number of iterations should be greater than 50k for 256 variables (i.e. a metamaterial comprised of 16 × 16 elements).

### Traps quality

In order to rate the quality of an acoustic levitation position (a.k.a. a “trap”), we locally approximate the Gor’kov potential with a quadratic form, thus considering the acoustic radiation force^1^ as a linear restoring one, with the elastic constant ${k}_{a}$ in the Cartesian direction $\overrightarrow{a}$. This approximation, which is only valid in the close proximity of a trap but commonly used to evaluate the strength of optical tweezers^2,55^, is now an accepted method also in acoustics^56–59^, where:

3 $${\tilde{k}}_{a}=\frac{{\partial }^{2}U}{\partial {a}^{2}}.$$

The value of ${k}_{a}$ can also be used to compare the stability of different traps, as large positive values of ${k}_{a}$ are linked to stronger attractive forces, while negative values denote instabilities.

**Figure 3:**
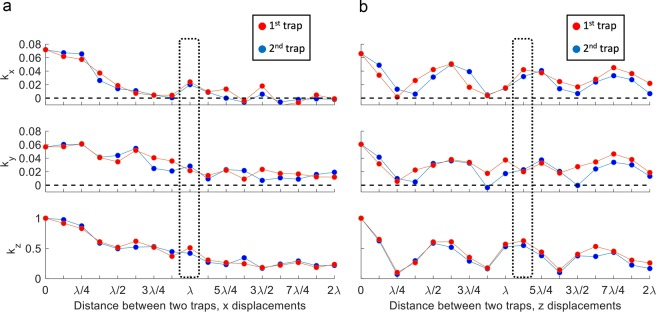
Calculated elastic constant in ${\rm{x}},{\rm{y}}$ and ${\rm{z}}$ directions for two levitation positions, obtained optimizing the displacements of a metamaterial with 16 × 16 elements opposing an 8 × 8 array of transducers as the trapping points are moving apart in (**a**) ${\rm{x}}$ horizontal direction and in (**b**) ${\rm{z}}$ vertical direction. Simulation results (50 k iterations) in these graphs were normalised to the maximum value i.e. the force in the vertical direction when the two levitation points coincide. The dotted line highlights the experimental conditions used in Fig. 5a,b.

To illustrate how this analysis works, we consider in Fig. 3 the case of a metamaterial comprised of 16 × 16 elements of $\lambda /2$ by $\lambda /2$ surface area each, positioned above a $8\times 8$ array of transducers (10 mm diameter, operating at 40 kHz), with its height datum above the source at $12\lambda$. We analyse in Fig. 3 the case of two levitation points, initially positioned in the same position at ${\rm{x}},{\rm{y}}=0$ and ${\rm{z}}=7\lambda$ and then moving apart from each other, either horizontally (Fig. 3a) or vertically (Fig. 3b), with a relative step of $\lambda /16$ in opposite directions (leading to a distance step between them of $\lambda /8$), up to a relative distance of $2\lambda$. Trapping conditions for two points were obtained by running the SA optimizer for 50k iterations, minimizing the objective function defined in Eq. (2) with the heights of the metamaterial’s elements (${z}_{dn}$) as decision variables.

As shown in Fig. 3, the elastic constant was found to be much greater in the vertical direction than in the horizontal one. This was anticipated, since the proposed setup is comprised of two opposing elements. Equally expected is the fluctuation of period $\lambda /2$ observed during the mutual displacement in the vertical direction, due to the variation of the potential along a wavelength.

The fact that the values of the elastic constant for both traps at every step in Fig. 3a are comparable highlights the importance of the standard deviation factor in the objective function Eq. (2), in order to ensure that the traps are of a similar quality (for more details refer to supplementary information, Section S5). Conversely, in Fig. 3b the differences between the two traps are more apparent and the solver does not always find a solution where both of the traps are of a similar quality. Equally interesting is the presence of some relative positions where the elastic constants become negative i.e. where the simultaneous presence of two traps is not possible. This could be because the optimizer failed finding the right metamaterial’s geometry or because it is not feasible to create two traps at the certain locations with the system’s physical limitations (i.e. signals’ directivity, number of transducers, geometry of the metamaterial, etc.). Finally, the simulations in Fig. 3a show that – within the proposed approximations – it is theoretically possible to levitate two objects at horizontal distance of less than $\lambda /2$ (for $\lambda \approx 8.4\,mm$), with a spring constant that increases as the distance decreases.

As shown in Fig. 3b, our simulations in the vertical direction confirm the results of a classical levitator i.e. for distances lower than $\lambda$ the best trapping position is at a distance of $\lambda /2$ between the particles. For larger distances, however, as illustrated in Fig. 3, the levitation conditions for two points can be achieved without their vertical distance being a multiple of $\lambda /2$.

### Experimental realization

To test our simulations, we used a commercial board comprised of an array of 16 × 16 ultrasonic transducers emitting at 40 kHz and turned on the middle 8 × 8 units (with the same phase). Like in the simulations, we used an opposing metamaterial comprised of 16 × 16 square elements with sides of $\lambda /2$ by $\lambda /2$ (approximately 4.3 mm by 4.3 mm). A similar choice for the size of the unit cells has been discussed by Memoli *et al*.^33^, who presented it as the maximum size allowed for spatial sampling, according to the Nyquist theorem^60^. Choosing the maximum size possible also reduced the manufacturing challenges in our case, but our method would apply in principle also to smaller elements. As levitation material, we used polystyrene balls of $2\pm 0.2$ mm in diameter, which approximately equals to $\lambda /4$.

**Figure 4:**
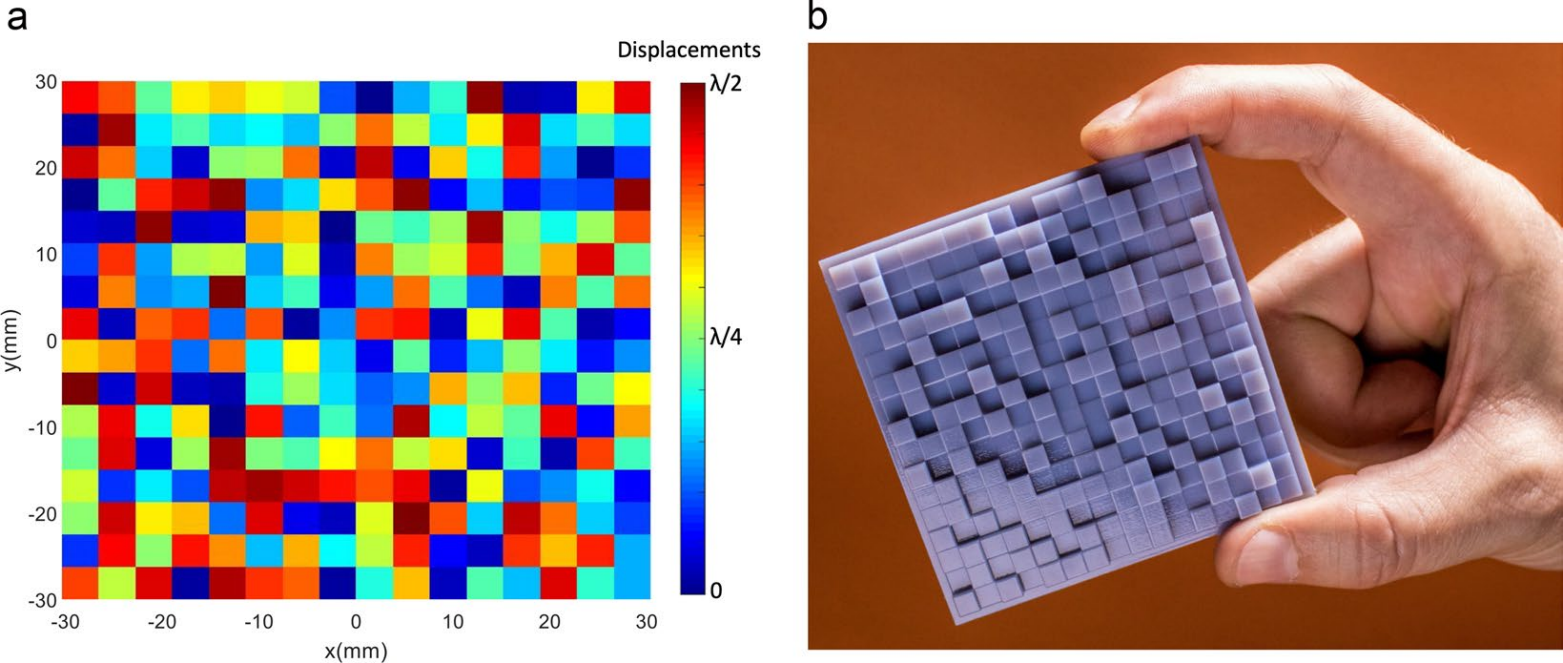
(**a**) The calculated grid of 16 × 16 elements metamaterial to fulfil the levitation requirements, (**b**) the relative 3D printed metasurface. Image (**a**) was created using MathWorks MATLAB R2018a.

After the algorithm computed an optimal set of displacements for the metamaterial (Fig. 4a), a 3D model of the metamaterial was created, and 3D printed (Fig. 4b). It is worth noting that, while the lateral dimensions of each element of the metamaterial can be as small as the fabrication apparatus (i.e. 3D printer) allows, as mentioned above each element was set to be $\lambda /2$ by $\lambda /2$ in lateral dimension and to have a maximum height relative to the datum of $\lambda /2.$ The maximum relative height was sufficient to cover the whole range of phase delays between 0 and $2\pi$ (see Methods).

**Figure 5:**
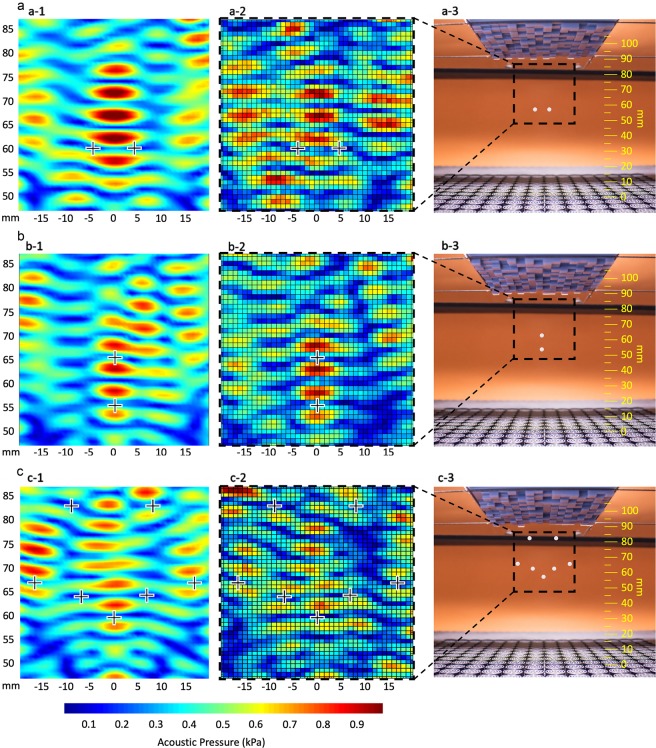
Acoustic levitation in predefined positions (crosses in **a–c** 1–2) with an array of transducers and an opposing metamaterial. (**a–c)** 1–2 the calculated and the measured pressure field in an ${\rm{xz}}$ plane at ${\rm{y}}=0$ and 3 a snapshot of the levitated beads. Levitation of **(a)** two beads at the same height with a horizontal distance of $\lambda$, **(b)** two beads at ${\rm{x}}=y=0$ with a vertical distance of $\lambda +\lambda /8$ and **(c)** seven beads forming a smiley face. Plots created using MathWorks MATLAB R2018a.

Considering the results illustrated in Fig. 3a, we initially investigated two traps separated horizontally, positioning them at distances ${\xi }_{x}\le \lambda$. By using polystyrene spherical particles of approximately $\lambda /4$ in diameter we did not manage to achieve stable levitation for distances smaller than $\lambda$ in between them at every direction: either the particles were attracted, collided and levitated at the same location or they were both ejected from the cavity. Our set-up could not distinguish between acoustic and electrostatic interactions – both neglected in our simulations – but acoustic inter-particle forces are the most probable cause for the collisions, since at distances greater than $\lambda /2$ they compare in intensity with the restoring forces in Fig. 3. Electrostatic forces (particularly strong with polystyrene) would therefore act mainly with $\lambda /4\, < {\xi }_{x}\, < \lambda /2$. In the rest of this work we therefore fix our horizontal mutual distance to $\lambda$: the smallest local maximum in Fig. 3a (see dashed line). This condition – demonstrated in Fig. 5a with simulations (5a-1), measurement (5a-2) and actual levitation (5a-3) – is a smaller distance than the one presented by Melde *et al*.^15^ and comparable to the highest horizontal precision achieved in a levitator so far^21^.

In our final example (Fig. 5c), we show how the fine control allowed by metamaterials can be used to display figurative information, like in the levitation of seven beads forming a smiley face. In future, our method could be used to transfer information between remote users: the transmitter will encode a shape of the acoustic field into a reflective metamaterial, which the receiver will be able to replicate using levitating beads on a different (but similar) array of transducers.

## Discussion

One of the key assumptions for a classical levitator is the presence of a planar wavefront in a certain region of space: a condition typically achieved by conducting experiments with a single transducer, in the far field. Recreating this condition elsewhere (e.g. closer to the source) may be challenging, even when the source is (like in our case) an array of ultrasonic transducers emitting all in phase. In our case, in fact, we did not manage a plane-wave emission even when treated surfaces were placed in front of the source to modify directly the emitted wavefront (see supplementary information,  S7).

Treating each transducer as an individual sound source overcomes this challenge, without the need of going in the far field^45,46^ or using only one transducer^20^ to obtain plane wave propagation, therefore opening the path to more compact or scalable devices. Using our method, in combination with feedback from measurements, it is possible to compensate in fact for any type and number of sources. Further, the computational method proposed holds for various systems where the variables to be optimized in order to create trapping points can be the phase and/or amplitude profile of PATs, the displacement profile of a grid of reflectors, the shape of reconfigurable metamaterials, the transducers and metamaterials topology or the relative positions of a mesh of particles to be levitated. We anticipate our generalized approach for acoustic levitation to enable new projects using multi-parametric systems and adopting either machine learning techniques or optimizing the proposed algorithm to run in real-time and create a powerful tool for synchronous manipulation.

The method described here also reduces drastically the need for the expensive and complicated electronics driving the transducers’ phases and amplitudes (PATs), as the user can choose the position of the levitation points a priori. We show how our algorithm can be used to encode complex information (here, a smiling face) into a reflective metamaterial, opening the way to displays that can easily be transferred from one user to another. Our method pushes the boundary of acoustic levitation, allowing for it a route to both scalability and miniaturization, moving the design limits for these systems to the resolution of 3D-printers. Although our algorithm cannot calculate an optimal set of parameters in real time, by pre-computing the relevant reflectors’ displacements, a dynamic grid of reflectors moving vertically^61^ could manipulate the levitated objects with high special resolution in 3D. Equally, future studies will consider further the case of hybrid levitators^62^, using a combination of PATs and (reflective) metamaterials.

Finally, the possibility of controlling acoustic fields using diffusers^63^ and source phase control^64^ is well-known in the audible frequency range. Considering that each levitation position is a local minimum of the pressure, our work shows that it is possible to create passively multiple regions where sound is much lower than in the local surroundings. Scaled at audible frequencies, our method may therefore lead to innovative ways of managing noise or to spatially immersive acoustic experiences.

## Methods

### Displacements limits

In order to minimize the implications, caused by the reflections on the side surfaces of the grid of the reflectors, the optimizer is constrained to displace the metamaterial’s reflecting surfaces between 0 and $\lambda /2$ (4.3 mm). A comparison between a model simulated with Finite Elements Method (FEM) that accounts for the side walls of the metamaterial and a model simulated with our equations, with a plane wave propagation (in order to simplify the 3D FEM model as our focus here was on reflections) and a random set of displacement for the metamaterial shows agreement within 10% (see supplementary information, S1). The distance between every reflector of the metamaterial to the control points in the cavity is not only in the vertical dimension and therefore, in order to enable the phase shifting of the reflected signal from 0 to 2$\pi$ the displacement ${z}_{dn}$ upper limit needs to be marginally extended. Nevertheless, because of the high directionality of the reflected waves, the most significant reflectors are the ones close to the vertical line joining the levitation position with the metamaterial’s datum. Therefore, the displacement limits do not need to be expanded.

### Derivatives

The derivatives were approximated by the numerical symmetric difference method, where the first order derivative of the function ${f}_{(x)}$, using Newton’s difference quotient, is^65^:

4 $${f}_{(x)}^{{\prime} }=\mathop{\mathrm{lim}}\limits_{h\to 0}\frac{{f}_{(x+h)}-{f}_{(x-h)}}{2h},$$

and for higher ($n$) order derivatives:

5 $${f}_{(x)}^{(n)}=\mathop{\mathrm{lim}}\limits_{h\to 0}\frac{1}{{h}^{n}}\mathop{\sum }\limits_{\alpha =1}^{n}{(-1)}^{\alpha +n}(\begin{array}{c}n\\ \alpha \end{array})\,{f}_{(x+ah)}.$$

In our code we set $h={10}^{-5}$.

### Experimental setup

We demonstrated acoustic levitation of multiple particles in predefined positions in a cavity between a single-phased array of transducers and an opposing reflective metamaterial. For all of the examples shown in Fig. 5 we used a commercial board (UltraLeap, version 2.0.0) where the central array of 8 by 8 transducers is emitting at maximum power at 40 kHz with zero phase shifting and an opposing metamaterial of 16 by 16 elements with a reflective area of $\lambda /2$ by $\lambda /2$ (4.3 by 4.3 mm) each, at a datum distance of 12 $\lambda$ (103 mm)from the array (the metamaterial’s elements are displaced towards the array). The measurements of the pressure field involved a high precision microphone moving mechanically with an automated system (see supplementary information, S8). We anticipate that the mismatch between the simulated and the measured pressure fields to be caused by the presence of the microphone in the cavity, as its diameter (~6.45 mm) is comparable to the sound field’s wavelength (~8.58 mm).

### Modelling assumptions

The equations used to simulate the pressure field^51^ allowed us to use a reflecting metamaterial at distances from the source closer than in previous studies, which implied a plane wave input^20,46^. The minimum distance between source and reflecting metamaterial allowed in this study is in fact $5\lambda$ (42.9 mm). We used the results by Silva and Bruus^27^ to evaluate the ratio between the inter-particle forces and the forces obtained by the Gor’kov formulation. For a sinusoidal acoustic standing wave varying in $\hat{z}$, this gives:

6 $$ratio=\frac{1}{2\pi }\cdot \frac{{(2\tilde{\rho }-1)}^{2}}{(2\tilde{\rho }+1)(5\tilde{\rho }-2)}\cdot {(\frac{{a}_{p}}{\lambda })}^{3}\cdot {(\frac{{\xi }_{z}}{\lambda })}^{-4}\,\sin \,[2\pi (\frac{{\xi }_{z}}{\lambda })],$$

where we assumed two spherical particles of similar radius (${a}_{p}$) at a mutual distance ${\xi }_{z}$ along $\hat{z}$, with $\lambda$ as the wavelength of the sound and $\tilde{\rho }$ as the ratio of the particle and fluid densities. With polystyrene spheres (density: 1052 ${\rm{kg}}/{{\rm{m}}}^{3}$) in air (density: 1.2 ${\rm{kg}}/{{\rm{m}}}^{3}$), we assumed $\tilde{\rho }\gg 1$.

## Supplementary information

Supplementary Information.

Supplementary Video.
