# Supplementary material for: Acoustic levitation with optimized reflective metamaterials
Source: Sci Rep. 2020 Mar 6;10:4254. doi: 10.1038/s41598-020-60978-4 (PMC7060201; doi:10.1038/s41598-020-60978-4)
Supplement: Supplementary file 2 — Supplementary Video. [file 41598_2020_60978_MOESM2_ESM.pdf]

Acoustic levitation with optimized reflective metamaterials

Spyros Polychronopoulos[1,2], Gianluca Memoli[1]

Affiliation: [1]University of Sussex, School of Engineering and Informatics, Brighton BN1 5EL, United Kingdom.
[2]currently at National and Kapodistrian University of Athens, Department of Informatics and Telecommunications, Athens, Greece.

Correspondence should be addressed to GM (e-mail: g.memoli@sussex.ac.uk), while requests for further material should be addressed to SP (email: spyrospoly@di.uoa.gr).

SUPPLEMENTARY INFORMATION

S1. Acoustic pressure calculation

We consider a set of ultrasonic transducers located at the plane $z = 0$ and a reflecting surface with a flat top reference plane (i.e. the "datum") located at $z = z_0$. The reflecting surface is made of $N_x N_y = N$ squared hard surfaces (i.e. the "reflectors"), each of them parallel to the $z = 0$ plane and placed at a different height respect to the datum. The centre of each of these surfaces is given by the vector $\boldsymbol{r}_\mathrm{n} = (x_n, y_n, z_n)$ expressed in Cartesian coordinates ($z_n \leq z_0$). We consider a single-frequency acoustic problem with angular frequency ω governed by the Helmholtz equation. The complex pressure at any point between $z = 0$ and z_0 can be calculated as the summation of the (direct) pressure created by the transducers, p_d, and the pressure reflected by the reflecting surface, p_r, reading

$$p(\boldsymbol{r}) = p_d(\boldsymbol{r}) + p_r(\boldsymbol{r}). \qquad \text{(S1)}$$

This equation implicitly neglects higher order reflections (i.e. reflections of the p_r on the transducers and subsequent reflections). As we shall discuss in section S2, these reflections are much smaller than p_d and p_r. Our analysis proceeds in three steps.

Step 1: Plane wave input, perpendicular incidence, single source.

First, we assume that the wave impinging on the surface of the metamaterial is a plane wave with a $\boldsymbol{k}$ vector $\boldsymbol{k} = k\,\hat{u}_z$, such that $p_d(\boldsymbol{r})$=$p_0 e^{i\boldsymbol{k}\cdot\boldsymbol{r}} = p_0 e^{ikz}$, where p_0 is a reference pressure and $k = 2\pi/\lambda$ is the wavenumber. In doing so, we omit any interference between reflectors. This approximation is equivalent to the one used by Melde[1] and Zhu[2] and is valid with at a sufficient distance from a generic source. The surface of each reflector is a hard boundary and, as such, the normal component of the total particle velocity on the surface of the reflector has to be zero. Thus

$$\left. v_{z,d} + v_{z,r} \right]_{z=z_n} = 0, \qquad \text{(S2)}$$

where $v_{z,\mathrm{d}}$ and $v_{z,\mathrm{r}}$ are the z-components of the particle velocity of the direct pressure and the reflected pressure, respectively. The particle velocity of the direct pressure can be easily calculated from p_d through the Euler's equation, which in the spectral domain reads

$$i\omega\rho_0 \boldsymbol{v} = \nabla p, \qquad \text{(S3)}$$

where ρ_0 is the density of the fluid between the source and the metamaterial (i.e. air, in our case). Then, $\left. v_{z,\mathrm{d}} \right]_{z=z_n} = \left. (i\omega\rho_0)^{-1} \partial_z p_d \right]_{z=z_n}$, and for the case of the plane wave, we find

$$v_{z,d}\big]_{z=z_n} = \frac{p_0}{c\rho_0} e^{ikz_n}, \tag{S4}$$

where c is the speed of sound in the fluid between the source and the reflector (i.e. air, in our case). It is worth to stress that, for the case of a plane wave travelling along z, v_z is the only component of the particle velocity different from zero, i.e. $v_{x,\mathrm{d}} = v_{y,\mathrm{d}} = 0$. By using the result of equation (S4) into equation (S2), we find that

$$v_{z,r}\big]_{z=z_n} = -\frac{p_0}{c\rho_0} e^{ikz_n}. \tag{S5}$$

We shall now calculate the complex pressure reflected by each of the reflectors by treating them as individual baffled square pistons. The far-field complex pressure distribution created by a rectangular baffled piston, $p^{(sp)}$, with centre at $\boldsymbol{r}_\mathrm{p} = (x_p, y_p, z_p)$ vibrating with a normal velocity v_z is given by[3]

$$p^{(sp)}(\boldsymbol{r}, \boldsymbol{r}_p) = \frac{-i\rho_0 c k a_x a_y}{2\pi} \frac{e^{ikd(r,r_p)}}{d(\boldsymbol{r}, \boldsymbol{r}_p)} v_z \, sinc\left(\frac{ka_x(x - x_p)}{2d(\boldsymbol{r}, \boldsymbol{r}_p)}\right) sinc\left(\frac{ka_y(y - y_p)}{2d(\boldsymbol{r}, \boldsymbol{r}_p)}\right), \tag{S6}$$

where $sinc(x) = \sin x / x$ while a_x and a_y are the corresponding side lengths of the piston and $d(\boldsymbol{r}, \boldsymbol{r}_\mathrm{p})$ is the distance between the points given by the vectors $\boldsymbol{r}$ and $\boldsymbol{r}_\mathrm{p}$, $d(\boldsymbol{r}, \boldsymbol{r}_\mathrm{p}) = \sqrt{(x - x_p)^2 + (y - y_p)^2 + (z - z_p)^2}$. By using equations (S5 and S6), the pressure reflected by the n-th reflector is

$$p_r^{(n)}(\boldsymbol{r}, \boldsymbol{r}_n) = \frac{ik a_x a_y p_0}{2\pi} \frac{e^{ikd(r,r_n)}}{d(\boldsymbol{r}, \boldsymbol{r}_n)} e^{ikz_n} \, sinc\left(\frac{ka_x(x - x_n)}{2d(\boldsymbol{r}, \boldsymbol{r}_n)}\right) sinc\left(\frac{ka_y(y - y_n)}{2d(\boldsymbol{r}, \boldsymbol{r}_n)}\right). \tag{S7}$$

In this equation, $d(\boldsymbol{r}, \boldsymbol{r}_\mathrm{n}) = \sqrt{(x - x_n)^2 + (y - y_n)^2 + (z - z_n)^2}$ is the distance between the centre of the n-th reflector and the point where the pressure is evaluated. This equation shows that the total phase acquired by the wave is $k(d(\boldsymbol{r}, \boldsymbol{r}_\mathrm{n}) + z_n)$, in agreement with the geometrical path described by the wave. The total reflected pressure is calculated as the superposition of the waves reflected by the N reflectors[4]

$$p_r(\boldsymbol{r}) = \sum_{n=1}^{N} p_r^{(n)}(\boldsymbol{r}, \boldsymbol{r}_n). \tag{S8}$$

Step 2: Generic wave, almost normal incidence, single source.

Next, we relax the plane-wave assumption and consider the direct pressure to be a generic wave $p_d(\boldsymbol{r})$. We also relax the conditions on incidence, assuming that the tangential velocity components at the surface of each of the reflectors are so much smaller than the normal one that can be neglected – i.e. "almost normal incidence" or $0 \approx v_{x,\mathrm{d}}, v_{y,\mathrm{d}} \ll v_{z,\mathrm{d}}$ at the n-th reflector. We maintain however that $v_{z,\mathrm{d}}$ is homogeneous across each reflector's surface. Our description corresponds to decomposing the direct wavefront into local plane waves $p_d = p_d(\boldsymbol{r_n})e^{ikz}$ in the close proximity of the reflecting metamaterial, where $p_d(\boldsymbol{r}_\mathrm{n})$ is the complex pressure evaluated at the centre of the n-th reflector. This approximation is valid closer to the source, but still in the far field (i.e. at distances above 5λ where λ is the wavelength of the direct wave).

$$p_r^{(n)}(\boldsymbol{r}, \boldsymbol{r}_n) = \frac{ik \, a_x a_y}{2\pi} \frac{e^{ikd(r,r_n)}}{d(\boldsymbol{r}, \boldsymbol{r}_n)} p_d(\boldsymbol{r}_n) sinc\left(\frac{ka_x(x - x_n)}{2d(\boldsymbol{r}, \boldsymbol{r}_n)}\right) sinc\left(\frac{ka_y(y - y_n)}{2d(\boldsymbol{r}, \boldsymbol{r}_n)}\right). \tag{S9}$$

where $p_d(\boldsymbol{r}_\mathrm{n})$ is the complex pressure evaluated at the center of the n-th reflector. The total complex reflected pressure can thus be evaluated substituting equation (S9) into equation (S8).

Step 3: Generic wave, multiple sources.

Finally, we consider the case of multiple sources. This step of the approximation is necessary to position our reflector closer to the source, but also because the pressure distribution created by the array of sources is usually far from being a plane wave, showing significant oscillations in amplitude and phase. As shown in section S3, this is true even when the source is composed by a phased array, with multiple transducers of the same type all vibrating in phase.

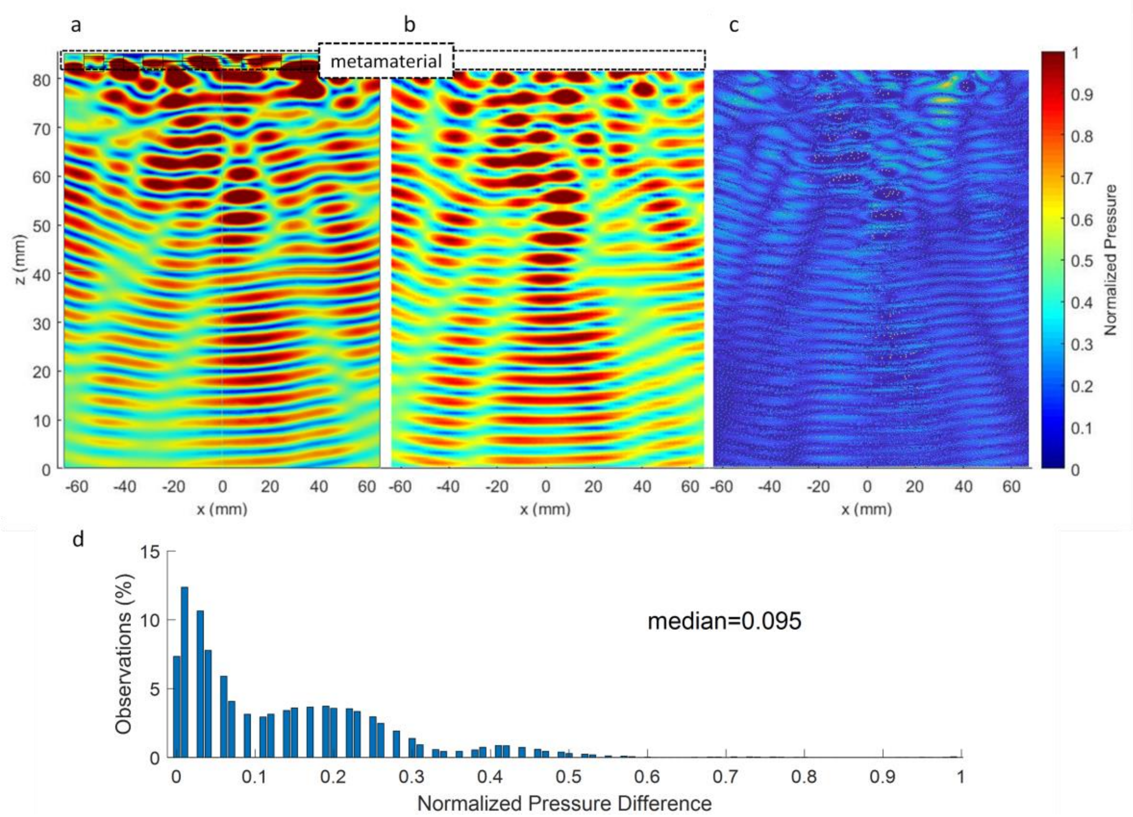

Figure S1 Benchmarking of our simulation technique. Plots a-b show the normalized sound pressure originated by a plane wave impinging on a reflective metamaterial with of 16x16 square elements of size $\lambda/2$, displaced from 0 to $\lambda/2$ from the datum at $z_0 = 10\lambda$: (**a**) 3D FEM model and (**b**) using equations (S1), (S9) and (S11). Plot (**c**) highlights the difference between the two methods (mismatch) and (**d**) reports the distribution of the normalized pressure difference between the two cases. Our median error is less than 10%.

In this work, we focus on the case of an array of circular transducers and we model each of them as a baffled circular piston. The far-field complex pressure created by the m-th circular piston is therefore given by[1]

$$p_d^{(m)}(\boldsymbol{r},\boldsymbol{r}_m) = p_0 \frac{J_1[ka_T \sin\theta(\boldsymbol{r},\boldsymbol{r}_m)]}{ka_T \sin\theta(\boldsymbol{r},\boldsymbol{r}_m)} \frac{e^{ikd(\boldsymbol{r},\boldsymbol{r}_m)}}{d(\boldsymbol{r},\boldsymbol{r}_m)}, \tag{S10}$$

where a_{T} is the radius of the piston, p_0 is a constant related to the power emitted by the transducer, $\boldsymbol{r}_{\mathrm{m}} = (x_m, y_m, z_m)$ is a vector denoting the position of the m-th transducer, J_1 is the Bessel function of the first kind, $d(\boldsymbol{r},\boldsymbol{r}_m)$ is the distance between $\boldsymbol{r}$ and $\boldsymbol{r}_m$, $d(\boldsymbol{r},\boldsymbol{r}_m) = \sqrt{(x-x_m)^2 + (y-y_m)^2 + (z-z_m)^2}$, and $\sin\theta(\boldsymbol{r},\boldsymbol{r}_{\mathrm{m}}) = \sqrt{(x-x_m)^2 + (y-y_m)^2} / d(\boldsymbol{r},\boldsymbol{r}_m)$. The total direct pressure generated by the array of N_T transducers is then calculated as

$$p_d(\boldsymbol{r}) = \sum_{m=1}^{N_T} p_d^{(m)}(\boldsymbol{r}, \boldsymbol{r}_m). \tag{S11}$$

It should be noted here that equation (S11) describes the pressure created by an array of transducers all emitting in phase. In order to account for a generic phase distribution of a PAT, the equation should include the phase shift φ_m of the m-th transducer and written as

$$p_d^{(m)}(\boldsymbol{r}) = p_0 \frac{J_1[ka_T \sin\theta(\boldsymbol{r}, \boldsymbol{r}_m)]}{ka_T \sin\theta(\boldsymbol{r}, \boldsymbol{r}_m)} \frac{e^{i[kd(r,r_m)+\varphi_m]}}{d(\boldsymbol{r}, \boldsymbol{r}_m)}. \tag{S12}$$

We benchmarked our model in different cases, comparing its predictions with the pressure simulated using a commercial Finite Element Method (FEM) package. One "worst-case" example can be found in Figure S1, where we considered a single sinusoidal plane wave (40kHz) impinging on a metamaterial's surface at $z_0 = 10\lambda$. The metamaterial is composed of 16x16 elements of $a_x = a_y = \lambda/2$. The 3D FEM model was build using COMSOL Multiphysics 5.3a Acoustic module, with air as propagation fluid and plastic for the metamaterial. The model using the equations (S1-S11) was simulated in MATLAB R2018a with the same set of displacements for the metamaterial's elements as in the COMSOL model. The xz plane plot at $y = 0$ of both the FEM model and our computational model Fig. S1a and S1b, respectively, and shows good agreement (see Fig. 1c and 1d, mismatch and distribution of the normalized pressure difference between the two fields). An analysis of the differences highlighted a median difference of less than 10% (i.e. 1 dB).

S2. Higher order reflections in the cavity

The normalized pressure fields created by the emitted, first and second order reflection signals are calculated and plotted in order to study their significance. We considered the reference absolute pressure value for the normalization to be the maximum one calculated field created by the emitted signals. Here, a system comprised by a 10mm in diameter transducer emitting sine wave at 40kHz and a flat reflecting surface of $a_x = a_y = \lambda/2$ at a distance of 2λ is simulated. The first and secondary reflections were simulated using the same method as described in the previous chapter in equations (S1-S11). For the second order reflections we considered a flat reflecting surface of $\lambda/2$ by $\lambda/2$ at the transducer's location. We have found that the pressure field created by the second order reflections' is approximately two orders of magnitude lower than the emissions' pressure field and therefore in our simulations were not taken into account. The calculated pressure profile in a perpendicular plane in the middle of the array (y=0) considering the signals from the transducer the 1st and 2nd order reflections, the transducer the 1st order reflections and the 2nd order

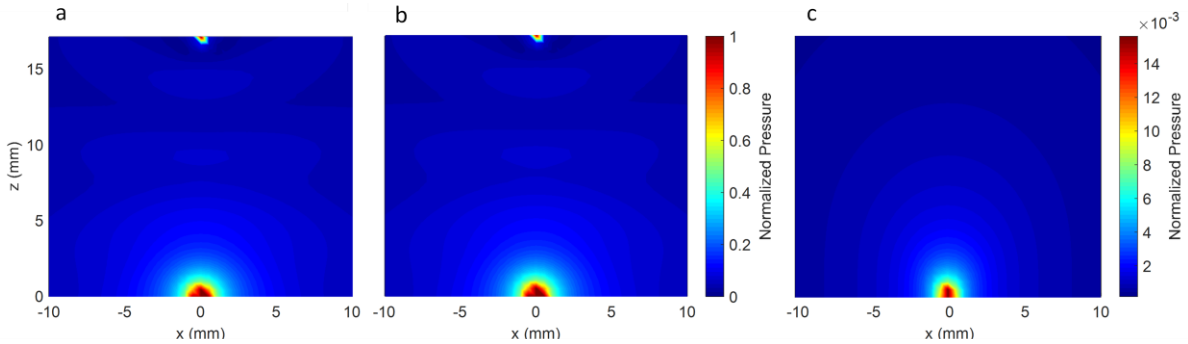

Figure S2 Signals comparison. Normalized sound pressure field in an xz plane at $y = 0$ of (**a**) The emission, first and second order reflections (**b**) the emission and first order reflection and (**c**) the second order reflection of 1 transducer 10mm in diameter at (x, y, z) = (0, 0, 0) emitting sine wave at 40kHz and opposing flat reflecting surface of an area of $\lambda/2$ by $\lambda/2$ at a distance of 2λ.

reflections are shown in Figures S2a, S2b and S2c, respectively. The amplitude of the reflected signals are significantly attenuated firstly because of the distance decay, as the travel path is longer than the direct path's,

and secondly because they are propagating, with a certain directivity, in all directions, as described in equation (S11). We find that taking into account the secondary reflections (Fig. S2a) does not significantly affect the pressure field comparing the pressure field without considering the second order reflections (Fig. S2b), as they are orders of magnitude lower in amplitude (Fig. S2c) than the source's amplitude.

S3. Radiation-force as a function of particle's size

Most of the literature on particle manipulation has assumed forces calculated according to the Gor'kov potential, which assumes non-interacting spherical particles with radius $a_p \ll \lambda$ (i.e. Rayleigh scattering conditions). Having discussed the role of interactions in the main text, we discuss here the role of particle size up to when $a_p \sim \lambda$ (Mie scattering), following the analytical model proposed by Silva *et al.*[5].

Assuming a 1D standing wave in the vertical direction, the radiation force is therefore given by:

$$F_{rad} = 2\pi a_p^2 \cdot Q_2 \cdot \frac{p_0^2}{4\rho_0 c^2} \cdot sin\, 2kz. \tag{S13}$$

The term Q_2 in equation (S13) is the "radiation-force efficiency" and, according to Silva *et al.*[5], can be calculated as:

$$Q_2 = -\frac{2}{(ka)^2}\sum_{n=0}^{\infty}(-1)^n(n+1)Im[S_n], \tag{S14}$$

where $S_n = s_n + s_{n+1}^* + 2s_n s_{n+1}^*$ (the asterisk denotes complex conjugation) and s_n is the scattering coefficient, given by:

$$s_n = det\begin{bmatrix} e_1 & d_{12} & d_{13} \\ e_2 & d_{22} & d_{23} \\ 0 & d_{32} & d_{33} \end{bmatrix} det\begin{bmatrix} d_{11} & d_{12} & d_{13} \\ d_{21} & d_{22} & d_{23} \\ 0 & d_{32} & d_{33} \end{bmatrix}^{-1}. \tag{S15}$$

The matrix elements[5] necessary to compute the scattering coefficient in (S15) are:

$e_1 = i\left({}^{ka_p}/_{\omega}\right) j_n'(ka_p)$,

$e_2 = -i\left({}^{\rho_0}/_{\rho_p\omega}\right) k_S^2 a_p^2 j_n(ka_p)$,

$d_{11} = -i\left({}^{ka_p}/_{\omega}\right) h_n^{(1)'}(ka_p)$,

$d_{12} = k_L a_p j_n'(k_L a_p)$,

$d_{13} = n(n+1) j_n(k_S a_p)$,

$d_{21} = i\left({}^{\rho_0}/_{\rho_p\omega}\right) k_S^2 a_p^2 h_n^{(1)}(ka_p)$,

$d_{21} = i\left({}^{\rho_0}/_{\rho_p\omega}\right) k_S^2 a_p^2 h_n^{(1)}(ka_p)$,

$d_{22} = -4k_L a_p j_n'(ka_p) + [(2n(n+1) - k_S^2 a_p^2)] j_n(k_L a_p)$,

$d_{23} = 2n(n+1)[k_S a_p j_n'(k_S a_p) - j_n(k_S a_p)]$,

$d_{32} = 2[j_n(k_L a_p) - k_L a_p j_n'(k_L a_p)]$,

$d_{33} = 2k_S a_p j_n'(k_S a_p) + \left[(k_S a_p)^2 - 2n(n+1) + 2\right] j_n(k_S a_p)$,

where $k_L = {}^{\omega}/_{c_L}$ and $k_S = {}^{\omega}/_{c_S}$ are the longitudinal and shear wave numbers inside the particle, with ρ_p and ρ_0 being the particle's and the medium's densities, respectively.

For our calculations we used polystyrene particles ($\rho_p = 1052\ \mathrm{kg\ m^{-3}}$, $c_L = 2400\ \mathrm{m\ s^{-1}}$ and $c_p = 1150\ \mathrm{m\ s^{-1}}$) in air ($\rho_0 = 1.2\ \mathrm{kg\ m^{-3}}$ and $c = 343\ \mathrm{m\ s^{-1}}$) with a frequency of 40kHz. In Figure (S3) we compare the force calculated using equation (S14) with the one calculated using the Rayleigh approximation[6], which gives

$$Q_2 = 2\,\Phi \cdot (k\,a_p), \tag{S16}$$

where Φ is the acoustophoretic factor ($\Phi \approx 5/6$ for particles in air).

Figure S3 shows that the actual force (calculated using Mie scattering) is typically smaller than the one calculated using Rayleigh conditions and much smaller already at $\frac{a_p}{\lambda} \approx 0.4$. In our experimental conditions (i.e. $\frac{a_p}{\lambda}$ ≈0.12) the difference is lower than 17%. We decided to neglect this difference, but a full optimization of the trapping field would need to take it into account.

It should be noted that levitation is still possible also at larger diameters: previous works succeeded even with larger particles $\frac{a_p}{\lambda}$ ≈0.17 (Pixiedust[7]) and $\frac{a_p}{\lambda} \approx$ 1.5 (Melde *et al.*[1]). As discussed in Silva *et al.*[5], however, larger diameters affect the stability of the traps.

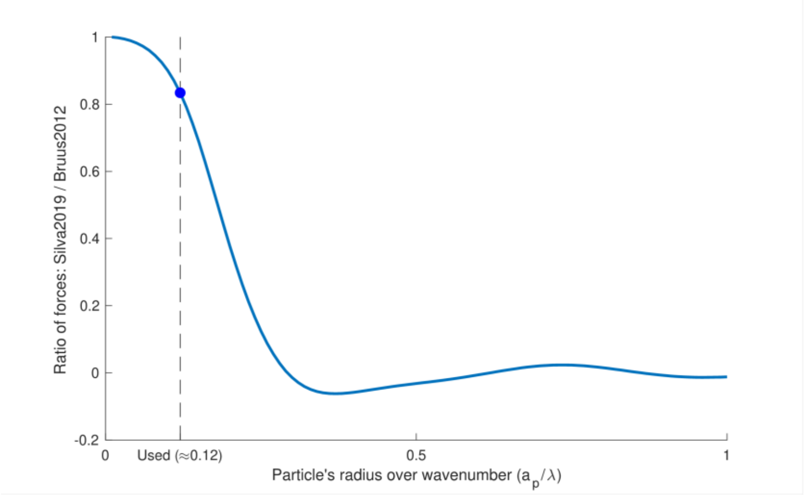

Figure S3 Effect of particle size on the acoustic radiation force for a 1D levitator. The plot reports the ratio between the force calculated under Mie scattering conditions (Silva et al., 2019) and the force calculated when the particle radius a_p is much smaller than λ (Rayleigh scattering according to Bruus 2012) for a polystyrene particle, levitated in air using 40 kHz (λ ≈8.6mm). The experimental data reported in this work are relative to $a_p/\lambda \approx 0.12$.

S4. Sound field created by an array of transducers

Here we plot the simulated (Fig. S4a) and the symmetric (symmetry line $x = 0$) measured (Fig. S4b), in xz plane at $y = 0$, sound fields created by an UltraLeap board, version 2.0.0. The board is comprised by an 16x16

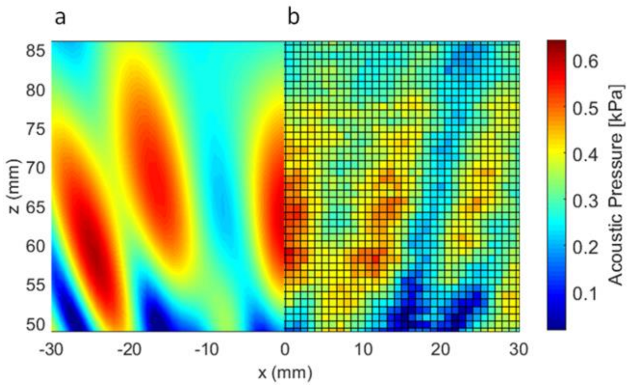

Figure S4 Plot of the pressure field created by an array of 8 by 8 transducers emitting synchronously sine waves at 40kHz in an xz plane at $y = 0$ showing (**a**) the simulated and the symmetrical (symmetry line x=0) (**b**) measured fields.

array of transducers (Murata MA40S4S) 10mm in diameter. Here we are using only the middle 8x8 array without phase shifting. The properties of each transducer were obtained from the manufacturer's manual, approximating each transducer as a cylindrical acoustic source emitting a sinewave at 40kHz with sound pressure level of 120dB at 30cm. The comparison is showing good agreement. Further, considering the simulation and the measurement results it is evident that the transmission is not comparable to a plane wave propagation.

S5. Numerical optimizer

The radiation force F^{rad} an acoustic field creates and it is acting on a small spherical particle (with radius $a_p \ll \lambda$) in an inviscid fluid can be calculated by[6]

$$F^{rad} = -\boldsymbol{\nabla} U^{rad}, \tag{S17}$$

where U is the Gor'kov potential which can be expressed as

$$U^{rad} = k_1|p|^2 - k_2\left(|p_x|^2 + \left|p_y\right|^2 + |p_z|^2\right), \tag{S18}$$

where $k_1 = \frac{\pi}{3}a_r^3\kappa_0(1-\tilde{\kappa})$, $k_2 = \frac{\pi a_r^3}{2\omega^2\rho_0}\frac{2(\tilde{\rho}-1)}{2\tilde{\rho}+1}$, a_r is the trap's radius, $\tilde{\kappa} = \frac{\kappa_p}{\kappa_0}$, with κ_p and κ_0 being the particle's and the medium's compressibility factors, respectively and $\tilde{\rho} = \frac{\rho_p}{\rho_0}$, and p_x, p_y and p_z are the first order derivatives of the pressure over x, y and z Cartesian coordinates, respectively. For air medium k_1 and k_2 can be simplified to $k_1 = \frac{\pi a_r^3\kappa_0}{3}$ and $k_2 = \frac{\pi a_r^3}{2\omega^2\rho_0}$, as $\kappa_p \ll \kappa_0$ and $\rho_p \gg \rho_0$.

The Simulated Annealing (SA) optimizer will output a set of displacement values for the metamaterial's grid of reflectors in order to create traps in the predefined positions. It is operating towards the minimization of the objective function with N displacement variables z_{dn}.

$$O = \sum_{j=1}^{J} O_j + e^{w_\sigma \sigma_O}, \tag{S19}$$

where J is the total number of traps, O_j is the objective function for trap j as described by Marzo A. et al.[8], σ_O is a function to ensure the similarity of the traps quality and it is the standard deviation of all the individual objective functions and w_σ is a weighting factor to enforce greater similarity. In our calculations we set $w_\sigma = 10$ but the value can be higher to enforce even higher trap similarity. Figure S5a illustrates the failure to create similar quality traps if the standard deviation is not considered. Whereas, expressing the objective function as in equation (S20) ensures that traps will have similar quality, Figure S5b.

$$O_j = w_{p,j}|p_m| - w_{x,j}U_{xx,j} - w_{y,j}U_{yy,j} - w_{z,j}U_{zz,j}, \tag{S20}$$

where aa subscript in the Gor'kov potentials denotes the second derivative in x, y and z. In order for the absolute value of the pressure to be comparable with the Gor'kov's derivatives we weigh the functions by w_p, w_x, w_y and w_z calculated by equation (S21). Following, by dividing by 3 the derivatives of the Gor'kov's potential – equation (S21) – we set the Laplacians' of the Gor'kov in all directions of equal importance and comparable to the absolute pressure. The values of the weighting factors are calculated by the mean values of a number of iterations of random set of displacements z_{dn}.

$$w_{p,j} = \frac{1}{\langle|p_j|\rangle}, w_{a,j} = \frac{1}{3\langle|U_{aa,j}|\rangle}. \quad \text{(S21)}$$

The implementation of the simulation and the optimization were both done in MATLAB and Global Optimization Toolbox Release 2018a. The algorithm systematically lowers the temperature reflecting to a finer search. SA reanneals after it accepts reanneal interval points. Reannealing sets the annealing parameters to lower values than the iteration number, thus raising the temperature in each dimension. The annealing parameters depend on the values of estimated gradients of the objective function in each dimension. For more information regarding the SA algorithm please see Ingber's articles[9,10].

Figure S5 shows an optimizer's performance example for 2500 iterations and 2 traps when the standard

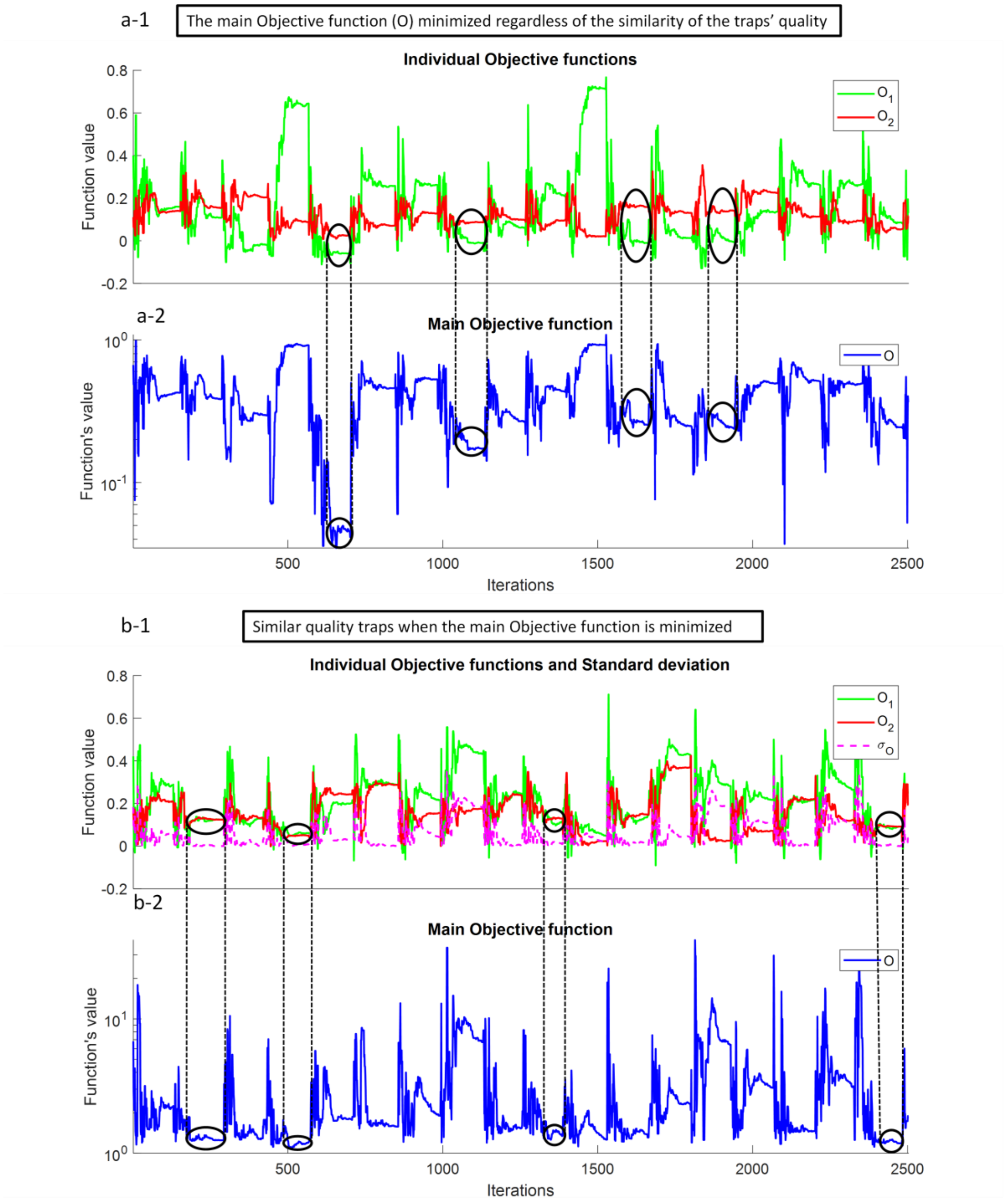

Figure S5 SA performance example for 2500 iterations and 2 traps when the objective function (**a**) does not include the standard deviation factor and (**b**) does include the standard deviation factor, is illustrating the objective functions of both traps and the standard deviation. (**a-1**) and (**b-1**) are showing the individual values of each objective function defining each trap's strength as a comparison with the main objective function show in (**a-2**) and (**b-2**).

deviation is not considered (Fig. S5a) and when the optimizer's objective function is O – i.e. see equation (S19) and Figure S5b. Figure S5 illustrates the values of both objective functions, O_1 and O_2, individually (green and red lines) and their standard deviation, σO, (yellow dashed line) in Figures S5a-1 and S5b-1 as SA is in operation minimizing the main objective function, O, (blue line) in Figures S5a-2 and S5b-2. When the objective function does not include the standard deviation factor - i.e. the second term of equation (S19) – the optimal solutions that SA provides does not ensure that all the traps are of a similar quality. As can be seen in Figure S5a the best cost function (first black circle) is when O_1 value is low but O_2 is much greater. When the objective function is the function defined in equation (S19) the main objective function O is minimized (second black circle in Figure S5b) only then O_1 value is comparable to O_2, making sure that the traps are of a similar quality. We have found that in order to ensure that the optimizer provides good set of displacements for two traps and for 256 variables (for example a metamaterial comprised of reflecting surfaces) the number of iterations should be typically more than 50k. The computational time for this number of variables and number of iterations was approximately 1.5 hours using a computer with Windows 7 operating system, 12 core 3.6GHz processor and 64GB of RAM. An animated visualisation of the optimisation process can be found in the supplementary video (sup_video_V1).

S6. Optimizing algorithm iterations' generated variables

In order to optimize the random reflectors' displacement values (z_{dn}) that SA generates at every iteration and more efficiently track down the optimum set of candidates, we applied a weighting factor to each reflecting surface. The algorithm considering the effectiveness of each reflector to each trap and then averaging the results:

$$W_N = \frac{1}{J}\sum_{j=1}^{J} W_j, \qquad \text{(S22)}$$

where $W_j = \begin{bmatrix} \sin\widehat{\beta_{J(1,1)}} & \cdots & \sin\widehat{\beta_{J(1,\sqrt{N})}} \\ \vdots & \ddots & \vdots \\ \sin\widehat{\beta_{J(\sqrt{N},1)}} & \cdots & \sin\widehat{\beta_{J(\sqrt{N},\sqrt{N})}} \end{bmatrix}$, β is the angle formed by the horizontal ray of the trap and the trap-center of the reflecting ray and the accent $\widehat{}$ denotes the normalization. The weighting, equation (22), is taken into account in order to optimize the generation of the random displacement values (z_{dn}) at every iteration of SA. The weighting factor describes the likelihood to change the previous randomly generated displacement value of each reflector and create a new set of random values at the next iteration.

S7. Treatment to recreate plane wave conditions

As discussed in the main text, the input condition of a propagating plane wave at normal incidence greatly simplifies our equations and, moreover, allows a direct comparison with literature results (e.g., obtained using a single transducer in the far field[1,2]). At the start of this study, we expected a plane wave at the output of a (16×16) speaker array, but our measurements found something else (Fig. S6a).

In this section, we describe our attempts to achieve plane wave transmission in front of a planar transducer array, where we mounted various perforated layers between the array and the receivers. If the holes in the perforated layer are much smaller than the wavelength, we thought, they should act as in-phase spherical emitters[4], adding up to a planar wave.

According to the fundamentals of acoustics, any source can be approximated by a spherical emitter when $kr_s \ll 1$, where k is the wave number and r_s the radius of the source[4]. In our case, where the transducers' emission frequency is 40kHz, source radius should be $r_s \ll 1.3\text{mm}$ for a spherical radiation. Thus, we micro-perforated (using a commercial laser cutter) a hard paper with 50% circle perforation and 0.15mm in radius holes to be placed on top of the array. The paper with no perforation is considered to be a reflective element as the measurement at 2cm above the array was 246 Pa and with the paper in between the array and the microphone was 21 Pa. It should be noted here that the heat of the transducers bent the perforated layer

when placed directly on top of them. Therefore, a wooden frame of 1cm thickness was used in three configurations. Blue: circular openings which they act as waveguides for each transducer d_1 equals the transducer's diameter with the perforated layer attached (see Fig. S6b), Red: square openings $d_2 = \lambda/2$ and 1mm walls with the perforated layer attached (see Fig. S6c) and Green: the perforated layer (see Fig. S6d).

As can be seen in the measurements of the sound field (Fig. S6) none of the treatments had a positive result. Further, in some cases (Blue and Red treatment) there was a great deal of sound power attenuation which is not in favor for acoustic levitation where strong sound field is required. The main reason that the treated surfaces were not effective is that the piezoelectric transducer's vibrating surface is not displaced as one piece and this is causing phase shifts on the input pressure on the holes of the perforated layer above.

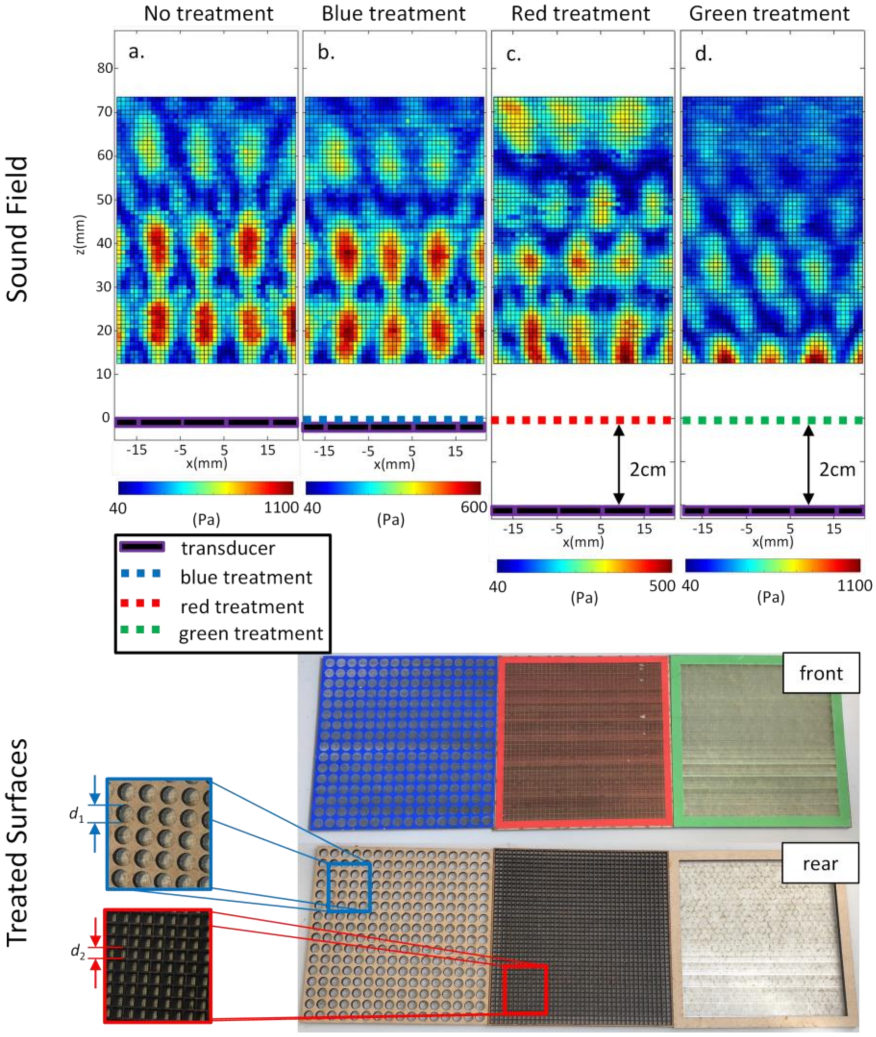

Figure S6 Comparison of the sound field created by a single phased array of 8x8 transducers without treatment and with three treated surfaces in order to approach a plane wave propagation. On the top a vertical cross-section measurement of the sound field in the middle of the array, the array of transducers and the treated surfaces are illustrated. On the bottom the front and the rear side of the treated surfaces (with a zoom detail) are shown. The sound field created by a single phased array of 8x8 transducers with **a.** no treatment, **b.** blue treatment i.e. waveguides on top of each transducer with a perforated layer attached, **c.** red treatment i.e. openings of $\lambda/2$ on with a perforated layer attached, **d.** perforated layer.

S8. Measurement system

In order to measure the sound fields generated, we built a custom 3D sound field scanner system (see Figure S7). We modified a commercial 3D printer (Velleman k8200), replacing its extruder by a fixed arm, holding a

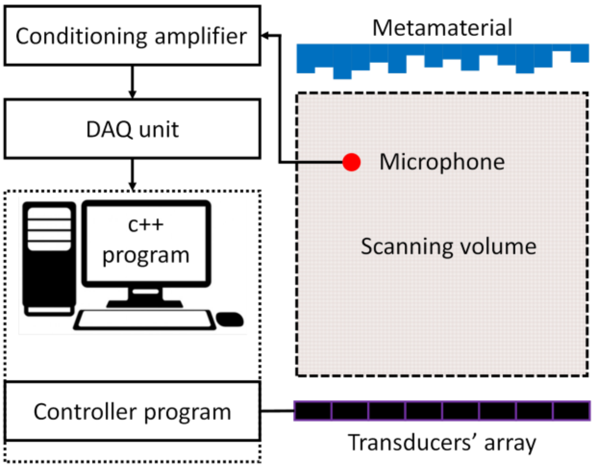

Figure S7 Measurement system showing the measurement and the levitation apparatus.

microphone. We then placed the board of transducers array (UltraLeap board, phased controlled using UltraLeap SDK, ver. 2.2.1) and the metamaterial at z_0 above the array. We set up this measurement system, ensuring the plate is parallel to the floor and the microphone is correctly aligned to the axis of the modulator (i.e. perpendicular to the board).

Finally, we used a custom-made C++ program, delivering G-code commands to the printer to control the position of the microphone, and take samples. A delay of 0.5s was included between the displacement end (steps of 1mm) and the sampling, to avoid displacement vibrations affecting the measurements. We used a 1/8-inch pressure-field B&K microphone (model 4138-A-015), a conditioning amplifier (Nexus Type 2692, final gain: -20dB) and a PicoScope data acquisition unit (DAQ), (Pico Instruments, model: 5444b), to capture samples and compute the sound pressure. The relative corrections in the measured amplitude due to the angle of the microphone in relation to the propagating acoustic wave (90°) were considered as the manufacturer specifies.

It should be noted here that as the microphone's diameter (~6.45mm) is comparable to the sound field's wavelength (~8.58mm) and therefore its presence in the cavity affects the field, in some positions more than others. We anticipate this effect to be the core factor for the mismatch between the predicted and the measured pressure field. Other less significant factors contribute to the mismatch between the simulated and the measured pressure field, as they were neglected in the modelling: 1) acoustic screening of the reflected waves, caused on the edges of the metamaterial elements; 2) higher-than-first-order reflections in the cavity, discussed in Section S2; 3) differences between the real emission of the transducers and the piston-like model – equation (S10) – as discussed in Sections S4 and S7; 4) cross-talking between different metamaterial elements, which is expected to be more relevant when the elements are smaller than $\lambda/2$ in size.

The distance between the levitated particles was measured by introducing a marked acoustically transparent metallic mesh in the cavity as the particles levitate.

—Video Caption—

Title: Dynamic visualisation of the optimisation process

Description:
The video shows the optimizer performing the iterations necessary to create two traps at a vertical distance of $\lambda+\lambda/8$. This video illustrates the two objective functions (one for each trap blue line, red line), the standard deviation between them (yellow line) and the main objective function (green line) for each iteration. To the right side, the video shows the metamaterial's elements (in purple) and the normalized acoustic pressure in the cavity with the two levitation points highlighted (blue dot, red dot) as the optimizer tries various displacements.

References

1. Melde, K., Mark, A. G., Qiu, T. & Fischer, P. Holograms for acoustics. *Nature* **537**, 518–522 (2016).

2. Hu, J. *et al.* Fine manipulation of sound via lossy metamaterials with independent and arbitrary reflection amplitude and phase. *Nat. Commun.* 1–9 (2018). doi:10.1038/s41467-018-04103-0

3. Williams, E. G. *Fourier acoustics*. *Book* (1999). doi:10.1016/B978-012753960-7/50004-8

4. Kinsler, L. E., Frey, A. R., Coppens, A. B. & Sanders, J. V. Fundamentals of acoustics. *Fundamentals of Acoustics, 4th Edition, by Lawrence E. Kinsler, Austin R. Frey, Alan B. Coppens, James V. Sanders, pp. 560. ISBN 0-471-84789-5. Wiley-VCH, December 1999.* **1**, 560 (1999).

5. Silva, G. T., Lopes, J. H., Leão-Neto, J. P., Nichols, M. K. & Drinkwater, B. W. Particle Patterning by Ultrasonic Standing Waves in a Rectangular Cavity. *Phys. Rev. Appl.* **11**, 54044 (2019).

6. Bruus, H. Acoustofluidics 7: The acoustic radiation force on small particles. *Lab Chip* **12**, 1014 (2012).

7. Ochiai, Y., Hoshi, T. & Rekimoto, J. Pixie Dust : Graphics Generated by Levitated and Animated Objects in. *ACM Trans. Graph.* **33**, Article 85 (2014).

8. Marzo, A. *et al.* Holographic acoustic elements for manipulation of levitated objects. *Nat. Commun.* **6**, 8661 (2015).

9. Ingber, L. Simulated annealing: Practice versus theory. *Math. Comput. Model.* **18**, 29–57 (1993).

10. Ingber, L. Adaptive simulated annealing (ASA): Lessons learned. (1995).
